# Circulating FGF21 proteolytic processing mediated by fibroblast activation protein

**DOI:** 10.1042/BJ20151085

**Published:** 2016-02-24

**Authors:** Eugene Y. Zhen, Zhaoyan Jin, Bradley L. Ackermann, Melissa K. Thomas, Jesus A. Gutierrez

**Affiliations:** * Tailored Therapeutics, Lilly Research Laboratories, Lilly Corporate Center, Indianapolis, IN 46285, U.S.A.

**Keywords:** dipeptidyl peptidase IV, fibroblast activation protein, fibroblast growth factor 21, post-transcriptional processing, prolyl peptidase

## Abstract

Fibroblast growth factor 21 (FGF21), a hormone implicated in the regulation of glucose homoeostasis, insulin sensitivity, lipid metabolism and body weight, is considered to be a promising therapeutic target for the treatment of metabolic disorders. Despite observations that FGF21 is rapidly proteolysed in circulation rending it potentially inactive, little is known regarding mechanisms by which FGF21 protein levels are regulated. We systematically investigated human FGF21 protein processing using mass spectrometry. In agreement with previous reports, circulating human FGF21 was found to be cleaved primarily after three proline residues at positions 2, 4 and 171. The extent of FGF21 processing was quantified in a small cohort of healthy human volunteers. Relative abundance of FGF21 proteins cleaved after Pro-2, Pro-4 and Pro-171 ranged from 16 to 30%, 10 to 25% and 10 to 34%, respectively. Dipeptidyl peptidase IV (DPP-IV) was found to be the primary protease responsible for N-terminal cleavages after residues Pro-2 and Pro-4. Importantly, fibroblast activation protein (FAP) was implicated as the protease responsible for C-terminal cleavage after Pro-171, rendering the protein inactive. The requirement of FAP for FGF21 proteolysis at the C-terminus was independently demonstrated by *in vitro* digestion, immunodepletion of FAP in human plasma, administration of an FAP-specific inhibitor and by human FGF21 protein processing patterns in FAP knockout mouse plasma. The discovery that FAP is responsible for FGF21 inactivation extends the FGF21 signalling pathway and may enable novel approaches to augment FGF21 actions for therapeutic applications.

## Abstract (precis)

Proteolytic cleavage of FGF21, evaluated in human plasma, confirmed three known proline hydrolysis sites. Although DPP-IV participates in consecutive cleavages from the N-terminus, fibroblast activation protein was implicated as the enzyme responsible for the C-terminal cleavage that inactivates FGF21.

## INTRODUCTION

Obesity and diabetes are part of a world-wide epidemic affecting millions of people. The collective burden imposed by these two chronic, and often related, diseases represents one of the major health and developmental challenges of the 21st century [1]. The prevalence of these diseases emphasizes the need for more efficacious options for drug treatment. Insulin, which was introduced almost a century ago, dramatically changed the treatment for diabetes, and still plays an important role in disease management. The pathobiology of diabetes is not homogenous and ranges from insufficient insulin synthesis to altered tissue sensitivity. Although co-morbidity between obesity and diabetes is well acknowledged, options for treatment are currently insufficient to treat this heterogeneous ailment.

Recent attention in the search for novel therapies has focused on fibroblast growth factor 21 (FGF21), a pleotropic metabolic regulator which has several potential applications in the treatment of metabolic disease [2]. Studies in obese rodents, diabetic rhesus monkeys [3,4] and human subjects with type 2 diabetes [5] have shown that systemically administered FGF21 may decrease blood glucose and triacylglycerol levels, improve insulin sensitivity and reduce body weight.

FGF21 belongs to the family of fibroblast growth factors consisting of 22 distinct members that interact with four distinct FGF receptors (FGFRs) on several tissues affecting tumour angiogenesis, wound healing, embryonic development and various endocrine signalling pathways [6]. FGF21 is a member of the FGF subfamily of ‘endocrine factors’ that comprises FGF19, FGF21 and FGF23. These three members require Klotho, a transmembrane protein, as a co-receptor to propagate intracellular signals. α-Klotho is required for FGF23 to regulate phosphate metabolism; and β-Klotho is required for FGF21 and FGF19 to regulate glucose/lipid homoeostasis and bile acid synthesis, respectively [7,8], by interacting through FGFR1 [9]. Immunoprecipitation studies showed that β-Klotho is constitutively bound to FGFR, independent of FGF21 [8]. Although FGFR is widely expressed, β-Klotho expression is limited to liver, pancreas and adipose tissue. The limited tissue distribution of β-Klotho probably determines the tissue specificity for FGF21 to mediate its metabolic activities.

Full-length human fibroblast growth factor 21 (hFGF21) is composed of 181 amino acids with a canonical ‘FGF-like’ domain. Studies with a series of truncated FGF21 proteins revealed that FGF21 interacts with β-Klotho through its C-terminus, and binds to FGFR1 through its N-terminus [10,11]. The C-terminal domain of FGF21 appears to be essential for enabling receptor/co-receptor mediated signalling. Removal of five residues from the N-terminus of FGF21 significantly reduced its signalling activity, and deletion of 10 residues from its C-terminus impaired its binding with β-Klotho, rendering it inactive [10,11].

In rodents and primates, the half-life of exogenously administrated hFGF21 is short, approximately 1–2 h [12–14], likely a result of enzymatic degradation and susceptibility to renal clearance. Indeed, FGF21 processing has been reported in earlier studies in preclinical species. During expression and purification in yeast, hFGF21 was found to be significantly proteolysed after the first four N-terminal amino acids [15]. When administered intravenously to mice or monkeys, hFGF21 was observed to be rapidly cleaved between Pro-171 and Ser-172 [12,16]. Furthermore, the relatively short half-life of the native protein in circulation makes it less ideal as a therapeutic agent, leading to significant efforts to mitigate this deficiency. Common half-life extension strategies have been explored for hFGF21, including the attachment of polyethylene glycol (PEG) to FGF21 [17–19], and the fusion of FGF21 to an Fc fragment [16]. These approaches significantly improved the pharmacokinetic properties of these FGF21 analogues *in vivo*; however, proteolytic processing still persists in these analogues. The introduction of site specific point mutations around Pro-171 was demonstrated to completely eliminate proteolytic processing after Pro-171 [16]. Critically, the ability to abolish FGF21 proteolytic cleavage by the introduction of a single point mutation suggested the involvement of a specific protease. Thus, identification of this protease may provide an alternative approach both to increase endogenous ‘active’ FGF21 levels and also to mitigate degradation of exogenous FGF21 via development of specific enzyme inhibitor.

In the present study, we report the processing of hFGF21 in human plasma and the detection of multiple forms of endogenous FGF21 proteins in circulation. Our results show that the circulating protease, fibroblast activation protein (FAP), is the proteolytic enzyme responsible for hFGF21 inactivation.

## EXPERIMENTAL

### Chemicals and reagents

Stable isotope-labelled (SIL) peptides were synthesized by CPC Scientific. Leucine residues were selectively labelled using ^13^C/^15^N-labelled amino acids resulting in the addition of 7 Da to the peptide mass (Supplementary Table S1). The reported purity of each synthesized peptide was more than 95% based on HPLC analysis. In addition, amino acid analysis (AAA) was performed to determine the peptide content for each standard and these values were used to establish concentrations used in quantitative studies. Protease inhibitor cocktail was purchased from Roche Diagnostics, dipeptidyl peptidase IV (DPP-IV) inhibitor was from LINCO Research. Aprotinin was from Fisher Scientific. Anti-FAP sheep polyclonal antibody and recombinant human FAP protein were from R&D Systems. Human and mouse FGF21 were generated in house as described in Micanovic et al. [10]. Reverse phase C18 Ziptips for small scale desalting were from EMD Millipore. Human FAP ELISA kit was from Adipo Bioscience. The FAP substrate [Suc-Gly-Pro-Leu-Gly-Pro-7-amido-4-methylcoumarin (AMC)] used in the kinetic assay was purchased from Bachem. Fluorescent AMC standard was purchased from BPS Biosciences. An FAP-specific inhibitor [20], a substituted 4-carboxymethylpyroglutamic acid diamide, was synthesized in house. A rabbit anti-FGF21 polyclonal antibody was generated in-house using the full-length human FGF21 as the immunogen. Mouse plasma from wild-type and FAP knockout mice was a kind gift from Dr E. Puré of the University of Pennsylvania [21].

### Human plasma collection

Human plasma was collected from seven healthy consenting donors with approval from the Lilly Multidisciplinary Program Oversight Committee. Blood (10 ml) was drawn from each donor into K_2_EDTA, P100 or P800 tubes (BD Diagnostics) depending on the experimental design. P100 tubes contain BD proprietary protease inhibitors, and P800 tubes contain DPP-IV inhibitors in addition to the protease inhibitors present in P100 tubes. Within 30 min of blood collection, the tubes were centrifuged at 1500 ***g*** for 10 min to collect plasma. Plasma was aliquoted and stored at −80°C prior to analysis.

Plasma samples (K_2_EDTA) from 48 donors were also purchased from Bioreclamation. Among the 48 donors, there were 22 females and 28 males, ranging in age from 40 to 70 years old and having body mass index (BMI) values ranging from 19 to 48.

### FAP depletion

Endogenous FAP in human plasma was depleted by immunoprecipitation using a sheep polyclonal anti-FAP antibody (R&D Systems). The antibody (200 μg) was immobilized to magnetic protein G Dynabeads (Life Technologies), and a bead aliquot containing 30 μg of immobilized antibody was spiked into 5 ml of human plasma. The sample was incubated with rocking at 4°C for 8 h or overnight. The beads were removed with the aid of a magnet, and another aliquot of beads was added to the sample for a second round of depletion. This process was repeated six times. Western-blot analysis performed after each round of immunoprecipitation showed that after the third round, no FAP was detected (data not shown).

### FGF21 *in vitro* digestion assays

For FAP *in vitro* digestion of FGF21, human or mouse FGF21 was incubated at 37°C with recombinant FAP (R&D Systems) at a ratio of 10:1 (FGF21:FAP) in digestion buffer (25 mM Tris, 0.25 M NaCl, pH 8.0). Aliquots were taken at different time points and analysed with an AB Sciex 5800 MALDI TOF mass spectrometer (AB Sciex) after desalting using C18 Ziptips.

### FGF21 *ex vivo* degradation in human plasma

Recombinant hFGF21 was spiked at 250 ng/ml into human plasma collected or prepared under different conditions (K_2_EDTA, P100, P800 or FAP depleted), and the samples were incubated at 37°C for 0, 24 or 48 h or longer. For studies with the FAP-specific inhibitor, the compound was added at 20 μM prior to incubation.

### FGF21 enrichment

FGF21 was enriched using an in-house made rabbit polyclonal anti-full-length FGF21 antibody coupled to Dynabeads M-280 Tosylactivated magnetic beads (Life Technologies) following the manufacturer's protocol.

To profile endogenous hFGF21, 5 ml of human plasma were used from each donor. Human plasma was first diluted 5-fold with dilution buffer (25 mM Tris, 25 mM HEPES, 300 mM NaCl, pH 7.5, 0.1% octylpyranoglucoside, protease inhibitor cocktail inhibitor, DPP-IV inhibitor and aprotinin), and 20 μl of antibody coupled beads were added. The samples were incubated overnight at 4°C with shaking. The supernatant was removed with the aid of a magnet, and the beads were washed three times using 1 ml of washing buffer (25 mM Tris, 25 mM HEPES, 500 mM NaCl, pH 7.5), followed by two washes with 1 ml of distilled water. The enriched FGF21 was either digested directly using trypsin, ASP-N protease or was eluted with 50 μl of 10% acetonitrile/0.2% TFA. For on-bead proteolytic digestion, the beads were suspended in 50 mM NH_4_HCO_3_, pH 9.0 for trypsin digestion or 50 mM Tris, pH 8.0 for Asp-N protease digestion. The samples were reduced first by adding 7 mM DTT followed by incubation at 55°C for 15 min. The samples were cooled to room temperature, and iodoacetamide was added to a final concentration of 15 mM. The samples were incubated in the dark for 15 min. Trypsin or ASP-N protease (0.7 μg) was added to the tubes, and the samples were incubated at 37°C overnight. Digests were acidified with a concentrated TFA solution, and FGF21 SIL peptides (100 fmol) were added as internal standards.

### FAP kinetic assay

A commercially available FAP substrate with the sequence Suc-Gly-Pro-Leu-Gly-Pro-AMC was used for FAP kinetic assessment [22], where Suc stands for succinyl and AMC for alpha-methylcoumarin. In each reaction with a total volume of 100 μl of solution, 20 μl of human plasma was added to assay buffer consisting of 20 mM Tris/HCl, pH 8.0, 0.1 M NaCl and 1 mM EDTA. The substrate was added to a final concentration of 20 μM. The samples were incubated at 37°C, and fluorescence was recorded using a microtiter-plate fluorometer (CytoFluro from Applied Biosystems) with an excitation wavelength of 360 nm and an emission wavelength at 460 nm. Serially diluted AMC fluorescence standards with known concentrations were also included to generate a standard curve for the product. FAP activity was calculated as the amount of AMC formed per minute per microlitre of plasma.

### Mass spectrometric analyses

Digested FGF21 peptides were analysed with a TSQ Vantage triple quadrupole mass spectrometer (Thermo Fisher Scientific) using a liquid chromatography-multiple reaction monitoring (LC-MRM) method specific for the targeted peptides. The peptides were separated using a Hypersil Gold C18 HPLC column (50 mm × 2.1 mm) with a Thermo Finnigan Surveyor autosampler and MS HPLC pump at a flow rate of 250 μl/min. Solvent A was 0.1% formic acid (FA) in water, and solvent B was 0.1% FA in acetonitrile (ACN). The HPLC gradient was as follows: 0–2.5 min, 5% B; 2.5–20 min, 5–34% B; 20–24 min, 34–70% B; 24–24.5 min, 70–80% B; 24.5–25 min, 80% B; 25–25.5 min, 80–85% B. The MS settings were as follows: capillary temperature, 300°C; vaporizer temperature, 274°C; sheath gas, 40; ion sweep gas, 0; aux gas, 25; spray voltage, 4000. The settings for individual transitions are listed in Table S2 of the supplementary material.

For MALDI-TOF MS analyses, an AB SCIEX TOF/TOF 5800 instrument (AB Sciex) was used with α-cyano-4-hydroxycinnamic acid (Sigma–Aldrich) as the matrix. All samples were analysed in linear mode using laser settings that do not induce in-source fragmentation.

To obtain the accurate mass of the FGF21 *ex vivo* digested products, the samples were also analysed using a ThermoFinnigan LTQ-FT mass spectrometer (Thermo Fisher Scientific). The samples were loaded on to a PicoFrit BioBasic C8 column (75 μm x 5 cm) (New Objective) using an Agilent 1100 nanoflow HPLC system run at 1 μl/min with 2% ACN/0.1% FA. After 10 min, the flow rate was changed to 250 nl/min for the duration of the run. The linear gradient was ramped to 80% of ACN/0.1% FA in 37 min. The electrospray voltage is 2600 V, and the outlet of the column was placed inside a modified Michrom ADVANCE source (Michrom Bio Resources) coupled to the mass spectrometer's inlet. Only MS scans obtained in the FT mode were collected. The MS data for the multiple charged ions were deconvoluted using in-house built software.

## RESULTS

### FGF21 degradation in plasma

**Figure 1:**
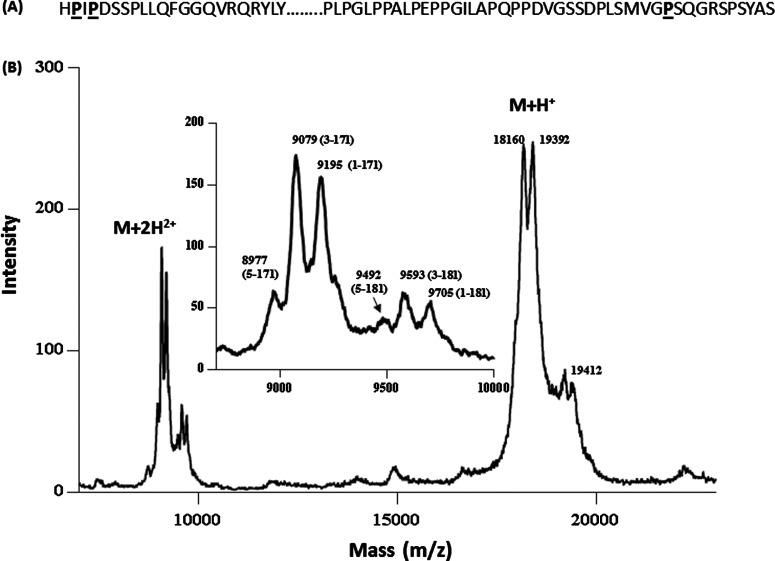
MALDI analysis of hFGF21 degradation in plasma (**A**) Human FGF21 sequence with proposed proteolytic residues highlighted; (**B**) human FGF21 was spiked into plasma at 3 μg/ml and was incubated at 37°C for 96 h. The degraded products were enriched using an anti-FGF21 antibody, and analysed using a MALDI-TOF instrument in linear mode. Both singly (M+H^+^) and doubly (M+2H^2+^) charged forms of the proteins were detected in the spectrum. The M+2H^2+^ forms were enlarged in the inset with the sequence information labelled in parentheses. The theoretical masses (in Da) for doubly charged species are as follows: FL: 9705; 1-171: 9195; 3-171: 9078; 5-171: 8977; 3-181: 9589; 5-181: 9483.

To determine the proteolytic processing of FGF21 in human plasma, recombinant hFGF21 protein was spiked into human plasma and incubated at 37°C for 96 h. The protein was subsequently enriched and analysed using a MALDI-TOF and an LTQ-FT mass spectrometer. As shown in Figure 1, after prolonged incubation in human plasma, the majority of the intact protein was degraded into several shorter forms. The mass difference between the shortest doubly charged species (M+2H^2+^, *m*/*z* 8977) detected and the intact protein (M+2H^2+^, *m*/*z* 9705) was 1456 (728 x2) Da, suggesting that hFGF21 was not cleaved in the middle of the protein, but more probably was processed close to the N- or C-terminus of the protein, resulting in a relatively small mass change. Both singly (M+H^+^) and doubly (M+2H^2+^) charged forms of the remaining intact and the degraded products were detected in the MALDI spectrum, and the doubly charged form provided higher resolution with more peaks resolved than the singly charged form. The mass difference between the intact protein and the degraded forms revealed that the major cleavages probably occur after Pro-171, Pro-2 and Pro-4 at the C- and N-termini, respectively. The same sample was also analysed by high resolution mass spectrometry (LTQ-FT MS), and accurate masses were determined for the intact and processed forms of hFGF21 (Table 1). The LTQ-FT data confirmed the assignments for the processed forms of hFGF21, revealing that, besides the presence of the intact (1-181) form, the major degradation products for hFGF21 are 3-181, 5-181, 1-171, 3-171 and 5-171. These results are consistent with the findings from several earlier studies showing that FGF21 cleavages occur primarily after Pro-2, Pro-4 and Pro-171 [15,16]. In the subsequent sections, human FGF21 forms truncated after Pro-2, Pro-4 and Pro-171 are referred to as FGF21 truncated after Pro-2 (ΔN2), FGF21 truncated after Pro-4 (ΔN4) and FGF21 truncated after Pro-171 (ΔC10), respectively.

**Table 1:** Accurate mass measurements for hFGF21 degradation products in human plasma after 96 h incubation at 37°C

		Selected ion		Averaged mass	
Sequence	Name	*m*/*z*	*z*	Theoretical	Measured
1-181	Full-length	1079.2735	18	19408.85	19408.83
1-171	ΔC10	1082.6155	17	18387.80	18387.40
3-171	ΔN2/ΔC10	1068.8451	17	18153.54	18153.37
5-171	ΔN4/ΔC10	1122.4511	16	17943.26	17943.17
3-181	ΔN2	1128.9309	17	19174.59	19174.80
5-181	ΔN4	1186.2879	16	18964.32	18964.60

To study the kinetics of FGF21 degradation, hFGF21 was spiked into plasma, with aliquots collected at different time points during incubation at 37°C. Exogenous hFGF21 was enriched through immunoprecipitation and proteolysed prior to MS analyses. The digested peptides were quantified using LC-MRM methods (N and C termini specific; Supplementary Table S2) with the corresponding SIL peptides added. Consistent with MALDI-TOF and high resolution LTQ-FT MS data, the major products detected using LC-MRM are the intact forms at both termini, ΔN2, ΔN4 and ΔC10 forms (data not shown).

To assess artefact proteolysis that may occur during sample collection and processing, plasma collected in various collection tubes (K_2_EDTA, P100 and P800) was spiked with exogenous hFGF21. The samples were incubated at 37°C for up to 48 h prior to immunoprecipitation and LC-MRM MS analyses. The hFGF21 degradation profile in P100 tubes was similar to control plasma without any protease inhibitor present (Supplementary Figure S1), suggesting that the protease inhibitors present in P100 tubes did not inhibit the protease(s) involved in FGF21 degradation. In contrast, the N-terminal processing of hFGF21 was diminished in the samples collected in P800 tubes (Supplementary Figure S1). This finding strongly suggested that a DPP-IV-like protease was likely to be involved in hFGF21 N-terminal processing. DPP-IV is a dipeptidyl peptidase (DPP) which preferentially cleaves Xaa-Pro or Xaa-Ala dipeptides from the N-terminus of a protein or peptide. Accordingly, the first four residues of hFGF21 contain two potential cleavage sites for DPP-IV allowing the ΔN2 and ΔN4 forms to be produced in sequential fashion (Figure 1). *In vitro* incubation of hFGF21 with exogenous DPP-IV also resulted in the rapid FGF21 processing to the ΔN2 and ΔN4 forms (data not shown). Together these data suggest that DPP-IV or a DPP-IV-like proteolytic activity is responsible for endogenous hFGF21 processing at its N-terminus.

### Endogenous FGF21 level and circulating forms

**Figure 2:**
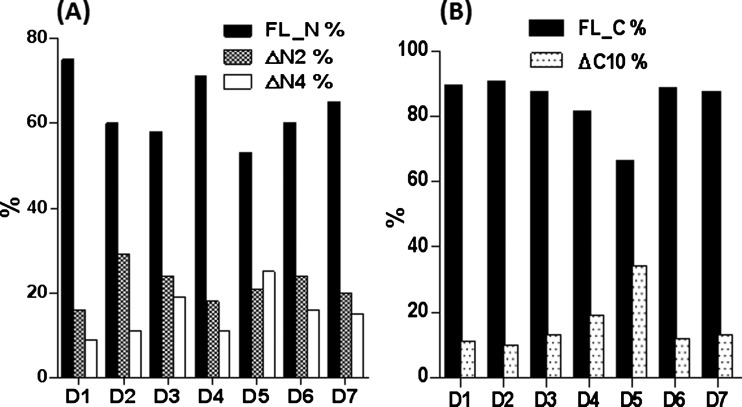
The levels of FGF21 processed products in healthy volunteer donors FGF21 from 5 ml of donor samples were enriched using an anti-FGF21 antibody, and analysed using LC-MRM. The percentages of the degraded products in each donor sample were plotted. (**A**) The N-terminal full-length (FL_N), ΔN2 and ΔN4 forms of FGF21; (**B**) the C-terminal full-length (FL_C) and ΔC10 forms of FGF21.

To establish the endogenous profile of FGF21 in humans, targeted LC-MRM methods were applied to plasma samples from healthy volunteers. Roche protease inhibitor cocktail and DPP-IV inhibitors were added shortly after K_2_EDTA plasma collection at room temperature**.** Separate samples with exogenous hFGF21 spiked at 20 ng/ml were used as positive controls. In seven donor samples, total FGF21 levels were found to be in the range of 0.1–0.45 ng/ml (Supplementary Figure S2), consistent with earlier reports. The total amount of hFGF21 in each sample was measured using either the N-terminal or the C-terminal peptide standards, with good correlation between concentrations determined using either method (Supplementary Figure S2). For control samples with 20 ng/ml of hFGF21 spiked-in, the measured concentrations were 24.2 and 25.7 ng/ml using the N-terminal and C-terminal methods, respectively. The N-terminal intact form of hFGF21 ranged from 50 to 75%, and the C-terminal intact form ranged from 70 to 90% in plasma samples from healthy humans. The cleaved forms for ΔN2, ΔN4 and ΔC10 ranged from 16 to 30%, 10 to 25% and 10 to 34% of circulating FGF21 levels in human plasma, respectively (Figure 2). Because of the precautions taken to limit *ex vivo* proteolysis during sample collection and processing, this distribution probably represents the relative endogenous levels of FGF21 forms in humans. Specifically, our data demonstrate that full length, ΔN2, ΔN4 and ΔC10 represent the major circulating forms of FGF21 in healthy volunteers.

### Fibroblast activation protein cleaves hFGF21 after Pro-171

The studies performed with DPP-IV inhibitors suggest that DPP-IV is likely to be the enzyme responsible for hFGF21 processing at the N-terminus, but not at the C-terminus. Because hFGF21 ΔC10 represents a significant percentage of circulating hFGF21 and also has been shown to be inactive *in vitro* [10,11], we considered the observed processing likely to be of functional importance and sought to identify the enzyme(s) responsible for cleavage at the C-terminal processing site.

**Figure 3:**
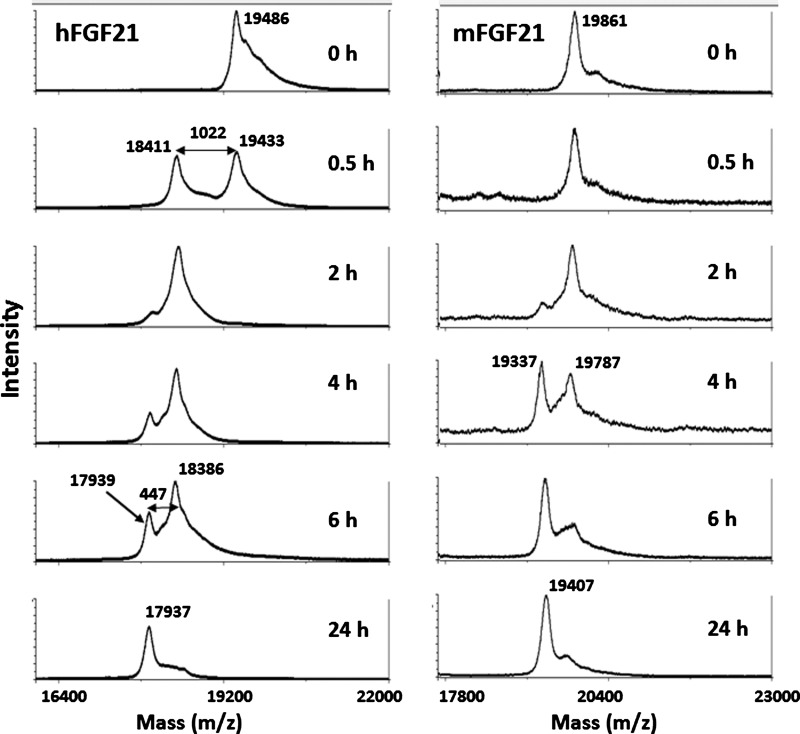
FAP digestion of human and mouse FGF21 Human or mouse FGF21 was incubated with human FAP at a ratio of 10:1 at 37°C. Aliquots were taken at different time points, and were analysed using MALDI-TOF mass spectrometry. Observed peaks in hFGF21 at *m*/*z* 19486/19433, 18411/18386, 17939/17937 correspond to full-length (1-181), 1-171, 5-171 of hFGF21, respectively; in mFGF21, *m*/*z* 19861/19787, 19337/19407 correspond to full-length (1-181), 5-181 of mFGF21, respectively.

Careful examination of the amino acid sequence at the three hFGF21 processing sites revealed all cleavages occur after proline residues (Figure 1). This sequence is suggestive of potential cleavage by prolyl peptidases, a family which includes DPP-IV. Three prolyl peptidases, prolyl endopeptidase [PrEP, also called prolyl oligopeptidase (PrOP)], lysosomal Pro-X carboxypeptidase (PrCP) and FAP (Seprase), were obtained and evaluated with regard to cleavage of both human and murine FGF21 *in vitro*. PrEP and PrCP did not cleave hFGF21 at the ΔC10 site even following prolonged incubation (data not shown). In contrast, FAP cleaved hFGF21 rapidly at several sites (Figure 3). The major peak (*m*/*z* 18411/18386) observed by MALDI-TOF appeared within 30 min following incubation with FAP. The mass difference between the full-length protein and this newly formed product was 1022 Da, close to the theoretical mass difference of 1021 Da between the full-length hFGF21 and ΔC10 form, suggesting that FAP is sufficient to cleave hFGF21 after Pro-171. The newly formed ΔC10 form was further processed sequentially to a form with an *m*/*z* of 17939. The mass difference between *m*/*z* 17939 and the ΔC10 form (*m*/*z* 18386) was 447 Da, consistent with the theoretical residue mass (445 Da) for the first four residues of hFGF21, suggesting that FAP alone can also process hFGF21 at the N-terminus of the protein to generate both ΔN4 and ΔC10 forms. The peptide sequence assignment for the FAP degradation products were further confirmed using LTQ-FT MS for accurate mass determination (data not shown).

As a prolyl endoprotease, FAP has a stringent amino acid sequence requirement (Gly-Pro) in its cleavage site requiring a proline residue at the P1 position and a glycine residue at the P2 position (P1 refers to the residue after which cleavage occurs and P2 is one residue N-terminal to P1) [23,24]. In hFGF21, the ΔC10 cleavage site contains a Gly-Pro sequence (Figure 1), making it a potential FAP cleavage site. In mouse FGF21, the corresponding glycine residue at position 170 is replaced with a glutamate residue, which should render the protein resistant to FAP cleavage. Indeed, when mouse FGF21 was incubated with FAP, ΔC10 cleavage was not observed, with only the ΔN4 product formed (Figure 3). There were no additional analyses for mouse FGF21 proteolytic processing in murine plasma.

**Figure 4:**
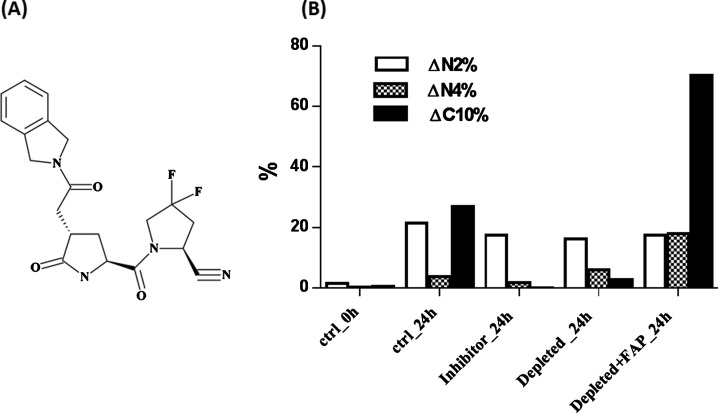
FGF21 processing in plasma with FAP inhibitor or FAP immunodepleted (**A**) Structure of FAP inhibitor. (**B**) FGF21 was spiked into control plasma (ctrl), control plasma with FAP-specific inhibitor spiked-in (inhibitor), FAP immunodepleted plasma (depleted) or FAP immunodepleted plasma with exogenous FAP spiked in at 200 ng/ml (depleted+FAP). The samples were incubated at 37°C for 24 h, and FGF21 cleaved products were quantified using LC-MRM.

To determine whether the endogenous circulating FAP enzyme in human plasma can process hFGF21 to the ΔC10 form, a previously reported FAP-specific inhibitor (IC_50_=22 nM) [20], a substituted 4-carboxymethylpyroglutamic acid diamide, was synthesized (Figure 4A), and spiked into control human plasma at 20 μM concentration. This inhibitor completely inhibited hFGF21 ΔC10 formation, while having a minimal effect on hFGF21 ΔN2 and ΔN4 formation (Figure 4B), suggesting that FAP or FAP-like activity is likely responsible for FGF21 ΔC10 processing in human plasma.

The involvement of FAP in hFGF21 cleavage was further evaluated through immunodepletion of plasma FAP. In these experiments, FAP in human plasma was depleted through several rounds of immunoprecipitation using an anti-FAP antibody. Human FGF21 was spiked into both FAP-depleted and control plasma, followed by incubation at 37°C for 24 h. FGF21 from both sets of plasma was subsequently enriched and digested, and the different forms were quantified using LC-MRM. In control samples after 24 h incubation, significant degradation of hFGF21 was detected at both the N-terminal and C-terminal ends, with FGF21 ΔN2, ΔN4 and ΔC10 forms accounting for 21, 4 and 27%, respectively (Figure 4B). Importantly, FAP immunodepletion reduced FGF21 ΔC10 formation to approximately 3%. In addition, FGF21 ΔC10 formation in the FAP-depleted plasma was rescued by reconstitution of the plasma with exogenous FAP enzyme, indicating that FAP was both necessary and sufficient for ΔC10 processing of hFGF21. The percentage of FGF21 ΔC10 formed reached about 70%, whereas the percentage of FGF21 ΔN4 formed also increased to about 18%, and the FGF21 ΔN2 level was relatively stable. These FAP inhibition and immunodepletion data implicate FAP as the enzyme responsible for ΔC10 processing of FGF21 in human circulation.

### FAP circulates in an active form in human plasma

To determine FAP concentrations in human plasma, K_2_EDTA plasma was collected from 48 donors, and FAP protein concentrations were measured using a commercial available ELISA kit. Among the 48 donors, FAP concentrations varied approximately 5-fold ranging from 50 to 250 ng/ml (Supplementary Figure S3). Among the donors, there were 13, 16 and 19 people with a BMI falling into the categories of Normal (19–24), Overweight (25–30) and Obese (>30), respectively. In each group, the FAP concentrations varied greatly, and no correlation between FAP concentration and BMI was observed (Supplementary Figure S4).

FAP activity was also measured for the 48 samples. The substrate used in the kinetics assay, a succinyl-pentapeptide composed of Suc-Gly-Pro-Leu-Gly-Pro-AMC, contains two FAP cleavage sites; however, only cleavage between the last proline residue and the AMC group releases the fluorogenic AMC group, resulting in a fluorescence reading. Under these specific experimental conditions, measured FAP activity of the samples ranged from 1.3 to 7 nM/min per μl. In the presence of an FAP-specific inhibitor, its activity was completely inhibited (data not shown).

The plasma FAP protein concentration and its activity showed good correlation (Supplementary Figure S3), suggesting that most FAP present in plasma is active, and that activity measured using this substrate is mainly from FAP.

### FAP is solely responsible for FGF21 cleavage in mouse plasma

**Figure 5:**
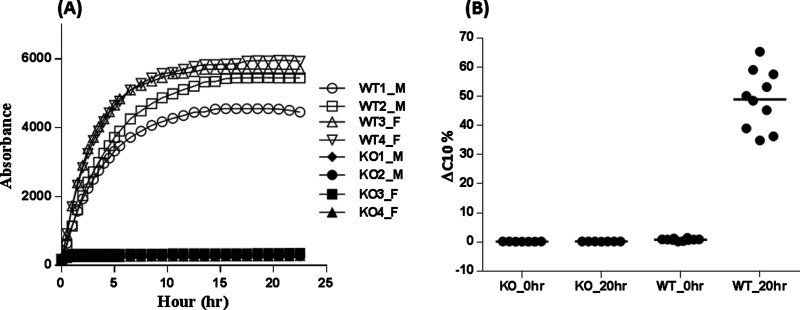
Human FGF21 cleavage in mouse plasma from wild-type and FAP knockout mice (**A**) FAP activity in plasma from wild-type (WT) and FAP knockout (KO) male (M) and female (F) mice. Two different samples from each gender were analysed for both the wild-type and the FAP knockouts; (**B**) human FGF21 ΔC10 cleavage in wild-type and FAP knockout mouse plasma after 20 h incubation.

Experimental evidence suggests that FAP can cleave hFGF21 after residue Pro-171 to generate the ΔC10 form of hFGF21, and that in human plasma this activity is mainly accounted for by FAP. However, whether FAP is the principal enzyme *in vivo* responsible for this activity is still unknown. To test this hypothesis, plasma samples from wild-type and from FAP deficient mice were obtained. We then assessed the FAP's activity of these samples using the assay described above. As shown in Figure 5, robust activity was obtained for wild-type mouse plasma independent of gender; however, no activity was detected in the FAP knockout plasma, consistent with their genotypes. Human FGF21 was subsequently spiked into plasma from wild-type and FAP null mice. Samples were then incubated at 37°C for 20 h, and the extent of hFGF21 C-terminal cleavage was quantified. As shown in Figure 5, no hFGF21 ΔC10 cleavage was detected in the FAP null samples after 20 h incubation; whereas in the wild-type mice, on average, 50% of hFGF21 was cleaved at the C-terminal end. These observations demonstrate that FAP is principally responsible for hFGF21 inactivation at the ΔC10 position. Although no human FAP null samples are available for testing, the combined evidence from FAP immunodepletion and inhibition studies, together with the evidence from mouse FAP knockout study, suggested that in human plasma FAP is likely to be the major enzyme regulating FGF21 C-terminal processing and inactivation.

## DISCUSSION

FGF21 has emerged as a powerful pleotropic regulator of metabolic homoeostasis and potentially represents a new class of therapeutics to treat diabetes and its co-morbidities. Administration of recombinant FGF21 to a wide array of animal models has demonstrated a number of therapeutic benefits including significant weight loss, enhanced glycaemic control and improved plasma lipid profiles [3,4]. Proof-of-concept human trials with optimized FGF21 analogues replicated the effects observed in earlier animal studies, demonstrating meaningful improvements in plasma lipid profiles and body weight reduction [5]. Although the preclinical efficacy of FGF21 demonstrated to date is impressive, the use of wild-type native FGF21 as a therapeutic agent is problematic due to several limitations, including a short half-life, significant proteolytic cleavage and rapid renal clearance. In the present study, we systematically studied the degradation of FGF21 and quantified the proteolytically processed forms in a small cohort of healthy human subjects. In addition, we demonstrated that DPP-IV is primarily responsible for FGF21 N-terminal processing, and that the circulating FAP is primarily responsible for the FGF21 C-terminal cleavage that deactivates the protein.

Basal levels of FGF21 in healthy adults have been measured to be within 50 pg/ml to 2 ng/ml using ELISA methods [25,26]. In a 7-day fasting study, FGF21 was observed to increase modestly [25]. FGF21 is known to be processed and cleared relatively rapidly with a half-life of 1–2 h when administrated to rodents or monkeys [12]. Depending on the site of cleavage, some products are inactive and do not bind to FGFR to elicit downstream signalling. Detailed analysis of FGF21 truncated proteins *in vitro* revealed that FGF21 N-terminal cleavage of up to four residues has no measurable effect on binding to FGFRs; however, deletion of six or more residues abolished FGF21 activity [10,11]. On its C-terminus, deletion of a few residues reduces binding to β-Klotho and lowers overall potency. Importantly, the ΔC10 truncated form of FGF21 has dramatically reduced binding affinity to β-Klotho when compared with full length protein, leading to almost complete loss of activity [10,11]. Most of the ELISA measurements of FGF21 utilize antibodies targeting the middle region of the protein, and probably measure ‘total FGF21’, including both active and inactive forms. Evaluation of all forms of circulating FGF21 is critical to understanding the efficacy of potential FGF21 therapeutics. To quantify both the intact and truncated inactive forms of FGF21, differential ELISA methodologies have been developed by using antibodies targeting different regions of the protein [12]. The endogenous FGF21 concentration measured from the small cohort in the present study and the percentage of C-terminal full-length protein determined using mass spectrometry is in general agreement with the results measured using our previously disclosed ELISA methodology [27].

In the present study, we have systematically studied hFGF21 degradation in plasma. Since endogenous FGF21 concentration tends to be low, exogenous hFGF21 was spiked into plasma and incubated for up to 96 h. The major cleavage occurs at the two terminal regions of the protein, after Pro-2, Pro-4 and Pro-171 residues, generating the so called ΔN2, ΔN4 and ΔC10 forms, which is consistent with the findings from several earlier studies [15,16]. In an earlier study with a FGF21-Fc construct [16], FGF21 was reported to be cleaved predominately after Asp-5 and Leu-21; however, we did not detect similar cleavages in the present study with hFGF21 alone. In the fusion protein of FGF21-Fc, the presence of Fc fragment probably changed the conformation of the FGF21 N-terminus, leading to protection from DPP-IV degradation. The results from studying exogenous FGF21 degradation helped us design a more targeted LC-MRM approach to focus on the N- and C-terminal regions of the protein. These methods were then used to characterize the endogenous forms of FGF21 in human plasma. The major FGF21 degradation forms *in vivo* were also found to be ΔN2, ΔN4 and ΔC10 forms. In a small cohort of healthy donors, the ΔN2, ΔN4 and ΔC10 FGF21 forms each account for around 10–35% of total FGF21.

Proline, unlike other amino acids, has a cyclic structure with its amine nitrogen bound to two alkyl groups, making it resistant to hydrolysis by many proteases. However, in the present study, we found that all three cleavage sites are after proline resides, suggesting that a specific class of proteases is probably involved in FGF21 processing. Prolyl peptidases, a sub-family of serine proteases belonging to the MEROPS peptidase family S9, can cleave after proline residues specifically. The members of the prolyl peptidase family include DPP-IV, FAP, DPP7, DPP8, DPP9, PrCP, PrEP (or PrOP) and acylaminoacyl-peptidase (AAP) [28].

Both DPP-IV and FAP are type II cell surface membrane proteins with the short N-terminal peptide domains as anchors to the external surface of the plasma membrane. In addition both proteins exist as a truncated soluble form in human plasma [29,30]. DPP7, DPP8 and DPP9 are also dipeptidyl peptidases capable of removing the N-terminal dipeptides provided proline is the penultimate residue. However, they are soluble proteins that have different subcellular locations than DPP-IV and FAP [31–33]. PrEP is a cytosolic enzyme that can cleave polypeptides in the middle of the sequence, and as its other name, PrOP, suggests, it is only active on short peptides up to approximately 30 amino acids long [34,35]. This would explain why FGF21 is not a good substrate for PrEP. The primary function of PrEP is in the maturation and degradation of peptide hormones and neuropeptides [36]. PrCP, on the other hand, cleaves the last C-terminal amino acid provided the penultimate residue at the C-terminal side is proline [34], which excludes the possibility that FGF21 is its substrate.

DPP-IV is the most extensively studied enzyme in this class of proteases. It removes the N-terminal dipeptides (Xaa-Pro or Xaa-Ala) from polypeptides provided that the penultimate residues are proline or alanine. One well-studied physiological substrate for DPP-IV is GLP-1, and the rapid cleavage of GLP-1 by DPP-IV renders GLP-1 inactive [37].

FAP is another widely studied enzyme in the family, and is most closely related to DPP-IV by sequence homology. Like DPP-IV, it retains DPP activity capable of removing the N-terminal dipeptides provided proline is the penultimate residue; however, unlike DPP-IV, it also retains endopeptidase activity [24]. The endopeptidase activity of FAP has a stringent sequence requirement with a proline at P1 position and a glycine at the P2 position (Gly-Pro) in its recognition motif. Moreover, the endopeptidase activity of the enzyme is much higher than its DPP activity [23,24,38].

FGF21 N-terminus contains a sequence of HPIP, an ideal substrate for a DPP. Both DPP-IV and FAP are capable of cleaving two residues at a time resulting in the FGF21 forms ΔN2 and ΔN4. In human plasma, hFGF21 N-terminal cleavage can be inhibited by DPP-IV specific inhibitors, suggesting that DPP-IV is the principal enzyme responsible for the cleavages. Although FAP retains DPP activity, and is capable of *in vitro* N-terminal FGF21 cleavage, it is not likely to be involved *in vivo*, since 95% of all DPP activity in blood has been linked to DPP-IV [39].

Unlike DPP-IV, which is expressed ubiquitously, FAP expression in healthy tissue is limited, and it has been detected during mouse embryogenesis, suggesting a role in developmental processes [40]. However, the FAP knockout mouse appears normal with no developmental defects, suggesting it is not essential to the development process [40]. In contrast, FAP has been detected in more than 90% of human epithelial tumours [41]. Genetic deletion or pharmacological inhibition of FAP can inhibit tumour growth in mouse models, suggesting that FAP is implicated in tumorigenesis [21], and it has since become a target for oncology treatment [42]. Although FAP is widely studied, its protein substrate repertoire is poorly understood [43]. *In vitro* screening of DPP-IV substrates revealed that FAP, as a DPP, can efficiently cleave neuropeptide Y, B-type natriuretic peptide, substance P and peptide YY [44]; however, whether these neuropeptides represent endogenous substrates for FAP remains to be verified. The only known substrates for FAP's endopeptidase activity are type I and III collagens and α2-antiplasmin [45–47]. Collagen is a major component of extracellular matrix, and rich in Gly-Pro motifs. The degradation of extracellular matrix by FAP potentially enables tumorigenesis. FAP alone cannot cleave intact collagens; only after initial cleavages by matrix metalloproteinases, can collagens be further degraded to smaller peptides by FAP [47]. The cleavage of α2-antiplasmin by FAP converts the protein into a more potent inhibitor of plasmin, suggesting FAP also plays a role in wound healing. There are no reports describing FGF21 as a substrate for FAP.

The discovery of FGF21 as a natural substrate for FAP expands our understanding of the biological function of this protease. Our mouse FAP knockout data demonstrate that FAP is solely responsible for hFGF21 C-terminal cleavage. However, in mouse FGF21, a glutamate residue is located at position 170 before Pro-171 (Glu-Pro), which makes mouse FGF21 resistant to FAP cleavage at this site in both mouse and human plasma. With the recent discovery of a rare variant form of FAP in humans that is functionally inactive [48], the question of whether in humans FAP is solely responsible for hFGF21 processing after Pro-171 can be evaluated. Since the FAP cleavage site on FGF21 is located at the C-terminal region of the protein, which also interacts with β-Klotho, the association of FGF21 with β-Klotho will probably affect FAP cleavage of FGF21. *In vitro*, pre-incubation of FGF21 with β-Klotho at 1:1 ratio dramatically reduced FAP cleavage after Pro-171 (data not shown); suggesting that, *in vivo*, β-Klotho probably plays a role in determining FGF21 degradation.

Proteolytic cleavage of hFGF21 after Pro-2, Pro-4 and Pro-171 has been reported previously [12,16]. Furthermore, rational design studies led to the removal of the first four N-terminal residues and to mutation of Gly-170 or Pro-171 to other residues as a method to improve the pharmacokinetic profiles of the engineered FGF21 analogues [12,15,16]. With the discovery of FAP as the endogenous protease responsible for hFGF21 cleavage after Pro-171, and our understanding of FAP sequence requirement (Gly-Pro), we now have a better understanding of how these mutations improve the stability of hFGF21.

As stated previously, FAP and DPP-IV are closely related and share a similar active site. Many of DPP-IV physiological substrates have been identified, including GLP-1 and GIP. Both peptides stimulate insulin secretion. Inhibition of DPP-IV reduces the degradation of GLP-1 and GIP, and enhances insulin secretion. FAP and DPP-IV can homodimerize [49,50], and active FAP/DPP-IV heterodimers have been identified [51].

DPP-IV inhibition is an effective treatment for diabetes, and FGF21 has also shown to be effective in lowering glucose levels. With the discovery of FGF21 inactivation by FAP, and the fact that DPP-IV and FAP share a similar structure, it is conceivable that a dual FAP/DPP-IV inhibitor could be developed to regulate glucose levels by two different mechanisms to achieve better glycaemic control and weight loss.

## Abbreviations

BMI: body mass index
DPP: dipeptidyl peptidase
DPP-IV: dipeptidyl peptidase IV
FAP: fibroblast activation protein
FGF21: fibroblast growth factor 21
FGFR: fibroblast growth factor receptor
FL: full-length
hFGF21: human fibroblast growth factor 21
LC-MRM: liquid chromatography-multiple reaction monitoring
PrCP: lysosomal Pro-X carboxypeptidase
PrEP: prolyl endopeptidase
PrOP: prolyl oligopeptidase
SIL peptide: stable isotope-labelled peptide
ΔN2: FGF21 truncated after Pro-2
ΔN4: FGF21 truncated after Pro-4
ΔC10: FGF21 truncated after Pro-171
